# Psychiatric services in primary care settings: a survey of general practitioners in Thailand

**DOI:** 10.1186/1471-2296-7-48

**Published:** 2006-07-24

**Authors:** Manote Lotrakul, Ratana Saipanish

**Affiliations:** 1 Department of Psychiatry, Faculty of Medicine, Ramathibodi Hospital, Mahidol University, Bangkok 10400, Thailand

## Abstract

**Background:**

General Practitioners (GPs) in Thailand play an important role in treating psychiatric disorders since there is a shortage of psychiatrists in the country. Our aim was to examine GP's perception of psychiatric problems, drug treatment and service problems encountered in primary care settings.

**Methods:**

We distributed 1,193 postal questionnaires inquiring about psychiatric practices and service problems to doctors in primary care settings throughout Thailand.

**Results:**

Four hundred and thirty-four questionnaires (36.4%) were returned. Sixty-seven of the respondents (15.4%) who had taken further special training in various fields were excluded from the analysis, giving a total of 367 GPs in this study. Fifty-six per cent of respondents were males and they had worked for 4.6 years on average (median = 3 years). 65.6% (SD = 19.3) of the total patients examined had physical problems, 10.7% (SD = 7.9) had psychiatric problems and 23.9% (SD = 16.0) had both problems. The most common psychiatric diagnoses were anxiety disorders (37.5%), alcohol and drugs abuse (28.1%), and depressive disorders (29.2%). Commonly prescribed psychotropic drugs were anxiolytics and antidepressants. The psychotropic drugs most frequently prescribed were diazepam among anti-anxiety drugs, amitriptyline among antidepressant drugs, and haloperidol among antipsychotic drugs.

**Conclusion:**

Most drugs available through primary care were the same as what existed 3 decades ago. There should be adequate supply of new and appropriate psychotropic drugs in primary care. Case-finding instruments for common mental disorders might be helpful for GPs whose quality of practice was limited by large numbers of patients. However, the service delivery system should be modified in order to maintain successful care for a large number of psychiatric patients.

## Background

Studies show a serious shortage of mental health professionals, particularly of psychiatrists [1-3] in developing countries. Thailand is one such country. It is a lower middle-income country with a population of 62.3 million in 2002, 65% of whom reside in rural areas. In 2000, there were 180,252 practicing medical doctors in Thailand [4]. In 2004, there were only seven psychiatrists per million inhabitants and 55% of them were concentrated in Bangkok [5]. There are no psychiatrists available in a number of provincial general hospitals. General practitioners (GPs) therefore play a crucial role in the detection and treatment of mental illnesses. Recently there was a radical shift in the health financing system in Thailand that placed even more emphasis on the importance of GPs in mental health care.

Since the Thai government embarked on a program to provide universal health-care coverage in 2001, dramatic changes have occurred in the health care provision in Thailand [6]. The system covers all previously uninsured people and those from the lowest income groups – more than 30% of the population. Under this new scheme, people buy a card that entitles the holder to medical care and treatment for a fee of 30 baht (0.70 USD) per ambulatory visit or hospitalization [7]. This covers most care except some high cost interventions such as kidney dialysis and antiretroviral therapy. Funding is through a capitation from the government's tax revenue to health care facilities according to the number of local residents registered with each facility. Primary Care Units (PCUs: health centres and units set up in hospitals to provide primary care) have been established to provide basic or primary care to the people within the vicinity of their residence, with a linkage in a holistic manner as well as referral system to higher levels of health care facilities. A typical province will have five to seven district primary care networks, each led by a contractor unit with one or two networks in the provincial city [8]. In rural or semi-rural areas, a patient's first contact with medical services is usually at the primary care unit. No appointments are necessary for consultation. A typical visit lasts about 3–5 minutes. Patients are allowed to access specialist care only after approval from their GP who is responsible for the referral. People may bypass primary care to a tertiary provincial general hospital but then they have to pay the cost themselves. Consequently, patients with psychiatric disorders usually choose to see GPs in their assigned PCU instead of going directly to a mental hospital or to the psychiatric department of the provincial general hospital.

Psychopharmacologic treatments for psychiatric disorders have advanced greatly in recent years. There are various new effective drugs with fewer side effects. It is therefore necessary for GPs to gain more knowledge and skills in diagnosing and treating common mental disorders in order to maintain the standard of care for psychiatric patients that will be more prevalent in primary care during this health reform period.

The purpose of this study was to investigate the prevalence of psychiatric disorders from GPs' perspectives, the drugs used, as well as the problems and barriers in providing psychiatric services in Thailand.

## Methods

This study was carried out by cross-sectional postal questionnaire survey. Ethical approval for the project was obtained from Ethical Clearance Committee on Human Rights Related to Research Involving Human Subjects, at the Faculty of Medicine, Ramathibodi Hospital, Mahidol University. A list of current community hospitals was obtained from the Bureau of Health Service System Development, Ministry of Public Health. During August-September 2004, we sent questionnaires to hospitals on the list, which consisted of 143 hospitals with only one doctor and 525 hospitals with several doctors. To avoid a clustering problem in the data from hospitals of the latter group, we sent only 2 copies of the questionnaire to each hospital. A total of 1,193 copies of the questionnaire were sent.

### Instrument

The postal questionnaire for the study consisted of 5 parts:

i) The GP's demographic and practice characteristics

ii) GPs were asked to rate the prevalence of 3 groups of patients; patients with physical problems, patients with psychiatric problems and patients with both problems.

iii) Prevalence of common psychiatric diagnostic groups. We have provided a brief description for each diagnostic group according to DSM-IV. We used DSM rather than the ICD-10 Primary Care Version, which would seem to be a more appropriate classification for GPs [9] because psychiatry teaching in the undergraduate medical curricula in Thailand is based on the DSM system as most of the senior faculty were trained from the United States. Consequently, GPs in Thailand are not familiar with the ICD-10 psychiatric classification system.

iv) GPs were asked to estimate the prevalence of each diagnostic group by using a visual analog scale (VAS). The visual analog scale consists of a ten point straight line with "0" at the left end and "100" at the right end indicating the percentage of each psychiatric diagnosis of total patients examined. GP's confidence in recognizing each psychiatric disorder was assessed by using a Likert-type scale with five options, on which "1" indicates least confident and "5" indicates most confident.

v) GPs were asked to rate the extent that each barrier has affected the diagnosis of psychiatric problems by using a Likert-type scale ranging from 1 (strongly disagree) to 5 (strongly agree).

vi) GPs were asked to rate the percentage of each psychotropic drug group prescribed and to name common drugs prescribed in each group.

### Data analysis

Statistical analyses were performed using the SPSS statistical package. Frequency distribution, cross-tabulation and chi-square tests were used to analyze categorical data. Continuous data were analyzed using student's t-tests and analyses of variance (ANOVAs). The Pearson product-moment correlation was used to establish the relationships between the variables. The significance level was set at P < 0.05.

## Results

### Sample characteristics

**Figure 1:**
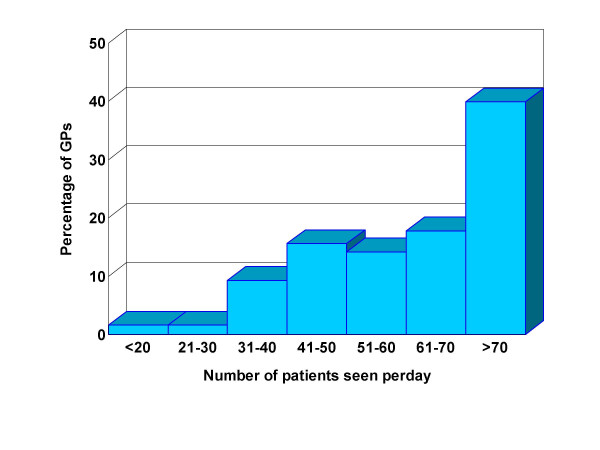
Percentage of GPs by consultation rates.

444 questionnaires were returned. Ten incomplete questionnaires were excluded. A total of 434 questionnaires were analyzable, giving a response rate of 36.4%. Sixty-seven of the respondents (15.4%) had taken further special training in various fields such as medicine, surgery and orthopedics. We excluded these from our analysis because their training might affect their perception and practice. As such, a total of 367 GPs were included in this study. Among these respondents, 56.4% were males and they had worked for 4.6 years on average (median = 3 years). The distribution of respondents by graduate medical schools was in line with that of the country. They practiced in all 5 regions of the country: Central (9.9%), East (9.02%), Northeast (35.5%), North (26.5%) and South (19.2%). Seventy-two per cent of GPs examined more than 50 patients per day and 40% of them saw more than 70 patients per day (Figure 1).

### GP's opinions on patients with psychiatric problems

GPs stated that 65.6% (SD = 19.3, median = 70) of their total number of patients examined had only physical problems, while 10.7% (SD = 7.9, median = 10) had only psychiatric problems and 23.9% (SD = 16.0, median = 20) had both physical and psychiatric problems.

Results of a one-way ANOVA showed a significant difference between the number of patients examined per day (<20, 21–30, 31–40, to >70) and the mean percentage of patients with psychiatric problems (F = 2.69; df = 6, 331; p < 0.015). A post-hoc Tukey test revealed a significant difference between GPs who examined 31–40 patients/day and those who examined 60–70 patients/day. There were no significant differences in the mean percentage of patients with physical problems and patients with both physical and psychiatric problems among each range of patients examined per day.

### Barriers to diagnosis of psychiatric disorders

Common barriers to diagnosis of psychiatric disorders were time constraints, lack of experience in psychiatric patient care and limited psychiatric knowledge (Table 1). For time constraints, the number of GPs who answered "agree" and "strongly agree" was as high as 76.2%.

**Table 1:** Barriers to diagnosis of psychiatric disorders

	Ranking of barriers to diagnosis (1–5, mean, SD)
Time constraints	4.1 (0.9)
Lack of experience in psychiatric patient care	3.7 (0.9)
Limited psychiatric knowledge	3.4 (0.8)
Lack of interviewing skill	3.4 (0.9)
Lack of interest in psychiatric services	3.1 (1.0)

A Pearson product-moment correlation was used to explore the association between each barrier problem and the perception of psychiatric prevalence. We found that the perceived psychiatric prevalence was significantly associated with only three variables. It had a negative correlation with the lack of interviewing skills (r = -0.136, P = 0.013) and the lack of experience in psychiatric patient care (r = -0.108, P = 0.048). However, it correlated positively with the problem of time constraints (r = 0.143, P = 0.009).

### Frequency of each psychiatric diagnosis

Table 2 shows frequently found diagnostic groups. These were anxiety disorders, alcohol and drug use disorders, and depressive disorders, respectively. GPs were more confident in assessing frequently found psychiatric diagnostic groups.

**Table 2:** Common psychiatric diagnostic categories and recognition confidence

	Per cent (SD)	Ranking of recognition confidence (1–5, mean, SD)
Anxiety disorders	37.5 (21.8)	3.5 (0.9)
Alcohol and drug use disorders	28.1 (19.6)	3.4 (0.8)
Depressive disorders	29.2 (20.0)	3.4 (0.8)
Psychotic disorders	23.9 (19.0)	3.3 (0.9)
Unspecified psychiatric disorders	21.0 (17.8)	2.4 (0.9)
Somatoform disorders	18.2 (16.8)	2.7 (1.0)
Organic mental syndrome	18.3 (15.5)	2.8 (0.8
Bipolar disorders	8.9 (8.2)	2.5 (1.1)
Child psychiatric problems	8.2 (9.7)	2.3 (1.1)

### Drug treatment

The most frequently used psychotropic drugs among GPs were anxiolytics, followed by antidepressants and antipsychotics, respectively. Frequently used drugs in each drug group were diazepam, amitriptyline and haloperidol, respectively (Table 3).

**Table 3:** Commonly prescribed psychotropic drugs by GPs

	% of total patients prescribed (SD)	Three most frequently prescribed drugs (percentage of each drug group)		
Anxiolytics	40.6 (20.5)	diazepam (76.7)	chlorazepate (10.3)	lorazepam (9.4)
Antidepressants	30.2 (19.8)	amitriptyline (94.5)	fluoxetine (4.0)	nortriptyline (1.5)
Antipsychotics	22.7 (17.7)	haloperidol (88.5)	chlorpromazine (9.4)	perphenazine (1.8)

## Discussion

According to Thai government regulations, newly graduated doctors have to start working up-country for 1–3 years before they can pursue further medical specialty training [10]. These physicians make up a substantial portion of the GPs in the country. Most of them worked in the Northeast, followed by the North which was close to the distribution proportion of GPs [4]. Overall, respondents' characteristics reflected the nature of GPs working in community hospitals.

Prevalence of mental disorder in primary care in developing countries varies from 9.8% in China [11], to 17–46% in India [11,12], 21.3% in Nigeria [13], 38.2% in Taiwan [14], 47–56% in Brazil [15] and 53.5% in Chile [11]. Thai GPs in this study perceived that about 10% of their patients had psychiatric problems, which was low compared to other studies. A major basis of this difference is that most of mentioned studies performed a direct assessment of the patients by using various instruments whereas our study was an opinion survey. Considering that there is a tendency of GPs to underestimate the prevalence of psychiatric morbidity among their patients [11,15], the actual prevalence of psychiatric disorders should be higher than what was found.

Our study revealed that GPs who faced with more time constraints tended to perceive that there were more psychiatric patients in their practice. It seems to contradict the commonly held view that the larger the number of patients examined, the more psychiatric problems go undetected and undertreated [16]. This is due to the fact that our study was an opinion survey. The answers of many respondents may be based on their attitude and concern but not their practice. Our findings may reflect that these GPs were concerned that there were a number of psychiatric patients in their routine care but they had difficulty in arriving at a definitive diagnosis due to time constraints. We are cautious in providing justification for this finding as the correlation between these two variables (the problem of time constraints and the perception of psychiatric problems) was very weak. A further study should be conducted to find whether their 'real world' practice shows the same tendency.

Our study showed that a number of patients with alcohol and drug abuse visited GPs. However, the questionnaire did not ask in detail about patients' alcohol related problems. In the international study of mental illness in primary care it was also found that alcoholic use disorders were a major problem in primary care of many countries [11]. A recent epidemiological study of psychiatric disorders in Thailand revealed that the prevalence of alcohol use disorders among community samples was as high as 28.5%, whereas the prevalence of major depressive disorder and generalized anxiety disorder were only 2.30% and 1.27%, respectively [17].

Our GPs perceived that their patients were more likely to have anxiety disorders than depressive disorders (37.5% vs. 29.2%) whereas studies from other countries showed that depressive disorders were much more common [11-13,15,18].

The reason that GPs perceived that there were not many patients with depressive disorders could come from that fact that GPs had less awareness of depressive disorders than anxiety disorders. A previous study of patients attending 4 sites of primary care setting in the central part of Thailand of Silpakit [19] showed that mixed anxiety and depressive disorder was much more common than depressive disorder. We did not have an item asking about mixed anxiety and depressive disorder in our study because it is a term in ICD-10 diagnostic categories that is unfamiliar to GPs. It is possible that GPs combined mixed anxiety and depressive disorder, which is less severe than major depressive disorder, with the anxiety disorder group.

Confidence in recognizing a disease was related to the frequency of disease found. However, this did not show that GPs' treatments were effective. For examples, GPs' confidence in treating depressive disorders was ranked high, but we found that their most frequently prescribed antidepressant was amitriptyline (94.5%) which has many adverse effects. A study conducted by Saipanish et al. 7 years ago [20], showed that, among antidepressants prescribed, 96.7% of GPs used amitriptyline. Therefore, it was only a slight decrease in amitriptyline use despite the fact that selective serotonin reuptake inhibitor (SSRI) antidepressants have been available in Thailand for more than a decade.

Current psychotropic drugs available in Thailand are not very different from those in many developed countries. Examples of these medicines are venlafaxine, mirtazapine, and escitalopram among antidepressant drugs; and olanzapine, ziprasidone, quetiapine, and aripiprazone among atypical antipsychotic drugs. However, these medicines are available only in university hospitals, mental hospitals, and large regional hospitals and they are not listed in the National Essential Drug List (NEDL). According to the government regulations, most medicines available at primary care are those listed in the NEDL. For medicines not listed in NEDL, patients have to pay out-of-pocket unless their physicians approve that the medicines as necessary and appropriate. For antidepressant drugs other than tricyclic antidepressants, mianserin and fluoxetine are the only SSRI included in the essential drug list. However, unfortunately, they are classified as secondary essential drugs that are not required to be available in every primary unit unless GPs requested for them. In most primary care units, only amitriptyline among antidepressant drugs and haloperidol among antipsychotic drugs are available whereas a few anti-anxiety medications such as diazepam, lorazepam and chlorazepate can be obtained.

Tricyclic antidepressants in primary care are frequently used in low doses that are unlikely to be of benefit in treating depression, generally because of their intolerable side effects [21,22]. Although there are arguments that low dosage tricyclic antidepressants are effective for depression, the low dose mentioned with these arguments is between 75–100 mg/day [23,24] whereas most GPs' in Thailand prescribed doses is between 25–50 mg/day.

Fluoxetine is a selective serotonin reuptake inhibitor (SSRI) antidepressant that has better tolerability and is easier to administer than tricyclic antidepressants [25,26]. Recent studies have shown that besides depressive disorders, SSRIs can also alleviate anxiety symptoms and treat some anxiety disorders such as panic disorder, social phobia and obsessive-compulsive disorder [27-29]. A generic form of SSRI such as fluoxetine is recommended as first line drug for the treatment of depressive disorders by the guideline from the National Institute for Clinical Excellence (NICE) [30] from England because it is as effective as tricyclic antidepressants and its use is less likely to be discontinued because of side-effects. It is also generally associated with fewer discontinuation symptoms. Recently, a study from Asian country has shown that fluoxetine can be used to treat patients with anxiety and/or depression safely and cost-effectively in primary care settings of low-income countries [31]. These advantages are reinforced by the fact that fluoxetine has been available in Thailand for more than a decade and there is now a generic version of fluoxetine available within the country at a very low price (0.05 USD per capsule). Attempts should be made to encourage GPs in our country to adopt fluoxetine as the first-line treatment for depressive disorders.

Anxiolytics were most frequently used among GPs in our study (40.6%). The high prevalence of benzodiazepines in primary care is common in most countries, especially the developing countries such as China (33%), India (32%) and Chile (48%) [11]. A previous study from Thailand found that, apart from psychiatric treatment, GPs also prescribed benzodiazepines for non-psychiatric conditions such as essential hypertension and uncomplicated lower back pain [32]. The most frequently prescribed anxiolytics in our study was diazepam, followed by lorazepam and chlorazepate. All of them are listed in the National Essential Drug List. A study by Srisurapanont et al. [32] showed that 45.5% of GPs stated that part of the reasons for their excessive use of benzodiazepines was the lack of time as well as the lack of knowledge and skills. This is in line with a study from Brazil which showed that GPs have a tendency to use benzodiazepines due to difficulties in making differential diagnosis [11].

Our study showed that psychiatric drug prescribing behavior by Thai GPs has largely been governed by administrative factors rather than academic progress. Evidence from developing countries suggests that common strategies such as disseminating treatment guidelines and providing training programs for GPs are only successful when they are accompanied by a strategy to increase the ease of accessibility to new and appropriate psychiatric drugs [11,33,34].

The perception of psychiatric prevalence among GPs in our study was low and the conditions of heavy workload left them with little time to provide a thorough assessment for psychiatric problems. Thus case-finding instruments for common mental disorders might play an essential role in assisting GPs in identify mental disorders in their patients. During the last few years, various screening instruments in Thai language have been developed to detect depression, anxiety and mental health conditions, such as the General Health Questionnaire (GHQ) [35], the Health-related self-report (HRSR) scale [36], the Thai Depression Inventory (TDI) [37], the Center for Epidemiological Studies Depression Scale (CES-D) [38] and the Hospital Anxiety and Depression Scale (HADS) [39]. However, new psychiatric cases found from such instruments may just be the add-on burden to GPs whose workload has been heavy and who are poorly equipped to manage the spectrum of disorders that presents to them. Fiscal and institutional constraints in health care services should be taken into account before adopting such service delivery programs in order to maintain successful care [40]. In this regard, lessons form developing countries might provide appropriate models. For instance, recently there was a report from Chile on the success of a program using multi-component intervention led by a non-medical health worker and included group psycho-education about depression, systematic monitoring of symptoms and a structured drug program for those with more severe depression [41]. In a region where there is a shortage of GPs, a structured approach that facilitates an increased role for non-medical staff, patients and family members may be more appropriate [42].

Certain limitations to this study should be recognized. Data from these questionnaires cannot reflect the nature of all GPs in community hospitals because a portion of GPs in community hospitals did not send back the questionnaires. These non-responding GPs might have been GPs who had only little interest in psychiatry or did not perceive it as a problem. However, because this study was a postal survey and the receiver could choose not to respond, we were unable to acquire further information from this group.

Another limitation is that, since our study was an opinion survey, it may not accurately indicate what doctors do in their 'real world' practice. The estimated percentage of each psychiatric disorder reflected what GPs perceived and practiced. However, some psychiatric disorders may have been under-detected or over-emphasized, especially anxiety disorders, depressive disorders, and somatoform disorders. Further study on the prevalence of psychiatric disorders in primary care settings and patients' chief presentations should be conducted to reveal a broader picture of psychiatric disorders in primary care in Thailand.

## Conclusion

Frequently found problems in primary care in our study were anxiety disorders, alcohol and drug abuse and depressive disorders. Most drugs available in primary care were the same as what existed 3 decades ago. The prescribing of benzodiazepines was common. Amitriptyline was a frequently used antidepressant although generic fluoxetine has been available for almost a decade. GPs' quality of practice was also limited by the large number of patients. Case-finding instruments for common mental disorders might help GPs in detecting common mental disorders in their patients. However, the service delivery system should be adjusted in order to maintain successful care for a large number of psychiatric patients

## Abbreviations

GP = general practitioner

VAS = visual analog scale

SSRI = selective serotonin reuptake inhibitor

## Competing interests

The author(s) declare that they have no competing interests.

## Authors' contributions

ML conceived and initiated the study, conducted the survey, analyzed the results and wrote the successive drafts of the manuscript. RS was responsible for completing the study, as well as for data entry, for assisting with data analysis and significant comments on the manuscript. Both authors have read and approved the final manuscript.

## Pre-publication history

The pre-publication history for this paper can be accessed here:


